# Association between the triglyceride-glucose-body mass index and the risk of early vascular aging in young and middle-aged Chinese adults: a cross-sectional study

**DOI:** 10.1186/s12872-026-06239-0

**Published:** 2026-09-25

**Authors:** Chaomin Kong, Caixia Lyu, Wenjin Lu, Xuedong Bai, Hongyang Dong, Feifei Zhang, Lixia Yao, Shuchun Chen

**Affiliations:** 1 Department of Internal Medicine, Hebei Medical University, No. 361 Zhongshan East Road, Shijiazhuang, 050017 China; 2 Department of Geriatric Cardiology, Hebei General Hospital, No. 348 Heping West Road, Shijiazhuang, 050051 China; 3 Examination Center, Hebei General Hospital, No. 348 Heping West Road, Shijiazhuang, 050051 China; 4 Department of Cardiovascular Medicine, Hebei General Hospital, No. 348 Heping West Road, Shijiazhuang, 050051 China; 5 Department of Endocrinology, Hebei General Hospital, No. 348 Heping West Road, Shijiazhuang, 050051 China

**Keywords:** Early vascular aging, Triglyceride–glucose–body mass index, Insulin resistance, Arterial stiffness, brachial–ankle pulse wave velocity, Framingham risk score

## Abstract

**Background:**

Early vascular aging (EVA) reflects accelerated arterial aging and is increasingly recognized as an important precursor of cardiovascular disease (CVD). Insulin resistance (IR) has been implicated in the pathogenesis of vascular aging. The triglyceride–glucose–body mass index (TyG-BMI), a surrogate marker of IR, has demonstrated predictive value for several cardiometabolic disorders. However, evidence regarding its association with EVA remains limited.

**Methods:**

This cross-sectional study enrolled 1,272 Chinese adults aged 30 ~ 59 years who completed brachial–ankle pulse wave velocity (baPWV) measurement. EVA was defined as a baPWV value above the 90th percentile in the corresponding 10-year age group. Multivariable logistic regression models were applied to assess the association between TyG-BMI and EVA. Restricted cubic spline (RCS) analysis was conducted to explore the potential nonlinear relationship. Additional analyses further examined the associations of TyG-BMI with baPWV and Framingham risk score (FRS)-derived indicators. Receiver operating characteristic (ROC) curve analysis was performed to evaluate the predictive ability of TyG-BMI for EVA.

**Results:**

After full adjustment, each 10-unit increase in TyG-BMI was associated with a 14.9% increased risk of EVA (odds ratio [OR] 1.149, 95% confidence interval [CI] 1.083~1.231). RCS analysis revealed a nonlinear association between TyG-BMI and EVA, with a turning point at a TyG-BMI value of 210.98. TyG-BMI also showed a nonlinear association with baPWV and FRS-derived indicators (predicted vascular age, age gap, and predicted 10-year CVD risk). In the fully adjusted model incorporating TyG-BMI, the area under the curve (AUC) for identifying EVA was 0.8533.

**Conclusions:**

This cross-sectional study demonstrated that TyG-BMI was independently and nonlinearly associated with EVA among young and middle-aged Chinese adults. The observed turning point suggests a potential reference point for this association. These findings suggest that TyG-BMI may serve as a simple, readily accessible indicator for assessing EVA risk in this population, pending external validation.

## Background

The global demographic transition toward an aging population has been accompanied by a marked increase in the prevalence of vascular aging–related conditions, including atherosclerosis, hypertension, and diabetes mellitus (DM) [1]. Vascular aging is characterized by progressive structural and functional deterioration of the vessel wall, primarily manifesting as increased arterial stiffness. This process is driven by endothelial dysfunction, elastin fragmentation, collagen deposition, and phenotypic modulation of vascular smooth muscle cells [2]. Such pathological remodeling underlies the pathogenesis of cardiovascular disease (CVD) and accounts for a substantial proportion of the global morbidity and mortality burden [3]. Epidemiological projections suggest that by 2030, vascular aging–related conditions will account for approximately 27 million cases of hypertension, 8 million cases of coronary artery disease, and 4 million cases of stroke worldwide [4, 5]. Consequently, early identification and effective management of vascular aging risk factors are imperative for the primary prevention of CVD and for alleviating the associated societal and economic burden.

Early vascular aging (EVA) refers to the premature deterioration of vascular structure and function, characterized by increased arterial stiffness and endothelial dysfunction, resulting in a vascular age that exceeds chronological age [6, 7]. Brachial–ankle pulse wave velocity (baPWV) has emerged as a practical, noninvasive surrogate for arterial stiffness, providing a quantitative measure of pressure wave propagation; higher baPWV values indicate greater arterial rigidity [8]. EVA was defined as a baPWV value above the 90th percentile within the corresponding 10-year age group [9, 10]. We applied the Framingham Risk Score (FRS) to calculate predicted vascular age, age gap, and vascular age, age gap (predicted vascular age minus chronological age), and predicted 10-year CVD risk. This scoring system incorporates conventional cardiovascular risk factors, including sex, age, high-density lipoprotein cholesterol (HDL-C), total cholesterol (TC), systolic blood pressure (SBP), smoking status, and DM [11]. Given the critical role of preventing EVA in mitigating future CVD risk, particularly among young and middle-aged adults, identifying reliable biomarkers for early detection is essential to guide timely intervention and inform personalized prevention strategies.

The triglyceride–glucose-body mass index (TyG-BMI), which combines the triglyceride (TyG) index with body mass index (BMI), has emerged as a practical surrogate marker of insulin resistance (IR) and has shown promising associations with hypertension, CVD, stroke, and mortality [12–15]. Compared with either TyG or BMI alone, TyG-BMI integrates information on both metabolic dysfunction and adiposity, and may supply complementary risk information [16]. Nonetheless, whether the composite indicator achieves significantly better discrimination than both the TyG index alone and BMI alone remains to be specifically verified in the context of EVA—an issue addressed in the current study.

Despite growing evidence linking TyG-BMI to adverse cardiovascular outcomes, its relationship with vascular aging remains largely unexplored. Given the central role of IR in arterial remodeling and vascular dysfunction [17], it is biologically plausible that elevated TyG-BMI may contribute to accelerated vascular aging. However, whether TyG-BMI is associated with EVA and whether this relationship follows a linear or nonlinear pattern remain unclear.

Therefore, the current study aimed to investigate the association between TyG-BMI and EVA in young and middle-aged Chinese adults. We further sought to characterize potential nonlinear relationships and assess the capacity of TyG-BMI to discriminate individuals at increased risk of EVA.

## Methods

### Data source and study population

This cross-sectional study was conducted at Hebei General Hospital and adhered to the ethical principles outlined in the Declaration of Helsinki. The study protocol was approved by the Ethics Committee of Hebei General Hospital (approval No. 2026-LW-126). Due to the retrospective and anonymized nature of the data, the requirement for written informed consent was waived by the Ethics Committee.

**Fig. 1:**
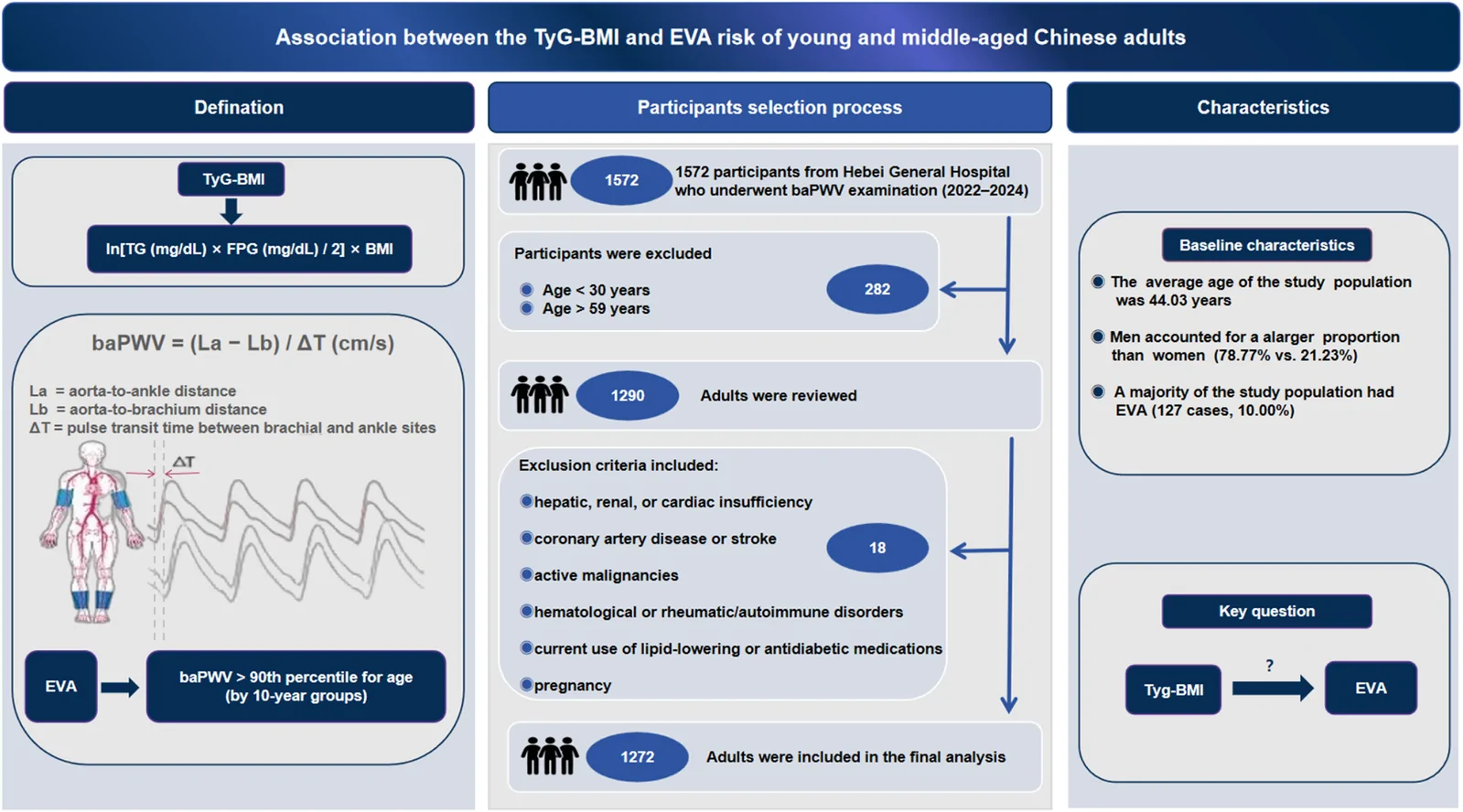
Scheme of the aim of the study and participants selection process. TyG-BMI, Triglyceride glucose-body mass index; EVA, early vascular aging; BaPWV, brachial-ankle pulse wave velocity

Participants were recruited from individuals receiving routine health examinations at Hebei General Hospital, representing a real-world screening cohort for early cardiometabolic risk assessment. This study initially enrolled 1,572 participants with available baPWV measurements from January 2021 to December 2024. Exclusion criteria were applied as follows: age < 30 or > 59 years; pre-existing hepatic, renal, or cardiac insufficiency; established coronary artery disease or stroke; active malignancies; hematological or rheumatic/autoimmune disorders; current use of lipid-lowering or antidiabetic medications; and pregnancy. No participants were excluded based on fasting plasma glucose or triglyceride outlier criteria. After all exclusions, the final analytical sample comprised 1,272 participants (Fig. 1).

### Data measurement and definitions

Demographic and clinical data were extracted from the hospital’s electronic medical records. Anthropometric parameters were measured using standardized protocols. BMI was calculated as weight (kg) divided by height squared (m²).Current smoking was defined as consuming ≥ 1 cigarette per day for ≥ 6 months. Similarly, current drinking was defined as consuming alcohol ≥ 2 times per week for ≥ 6 months. SBP was measured bilaterally, and the mean value was used for analysis.

After an overnight fast of ≥ 8 h, venous blood samples were collected. Biochemical parameters, including TC, triglyceride (TG), HDL-C, low-density lipoprotein cholesterol (LDL-C), fasting plasma glucose (FPG), glycated hemoglobin (HbA1c), and homocysteine (Hcy), were measured using a Beckman Coulter AU5800 automated chemistry analyzer. The TyG index was calculated as ln(TG × FPG / 2), with TG and FPG expressed in mg/dL. Since FPG and TG were measured in mmol/L, values were converted to mg/dL by multiplying FPG by 18.0 and TG by 88.6. TyG-BMI was subsequently derived as the product of the TyG index and BMI (TyG × BMI), serving as a composite continuous marker for IR.

Arterial stiffness was assessed using a Vascular Profiler BP-203RPE III (Omron, Kyoto, Japan), a validated non-invasive device for pulse wave analysis. Following a standardized protocol, participants rested in the supine position for ≥ 10 min to ensure hemodynamic stability. Four pneumatic cuffs were placed on both upper and lower limbs by trained technicians. BaPWV was calculated using the height-estimated path length and the pulse transit time between the brachial and ankle (posterior tibial) recording sites, with bilateral values averaged [18]. EVA was defined as a baPWV value exceeding the 90th percentile within the corresponding 10-year age group [9, 10].

Predicted vascular age and 10‑year CVD risk were estimated/derived from the FRS based on sex, chronological age, HDL‑C, TC, SBP (treated or untreated), smoking status, and diabetes status [11]. Age gap was calculated as the difference between predicted vascular age and chronological age. These three metrics served as secondary exploratory outcomes. To ensure computational stability, extreme predicted vascular age values (< 30 or > 80 years) were winsorized to 30 and 80 years, respectively [19].

### Statistical analysis

Statistical analyses were performed using R software (version 4.5.0), IBM SPSS Statistics (version 27.0), and GraphPad Prism (version 9.5), with a two-sided significance level set at *P* < 0.05. Participants were categorized into EVA and non-EVA groups based on baPWV thresholds (detailed in the Methods section). Baseline characteristics were compared between the two groups. Continuous variables are presented as mean ± standard deviation (SD) or median (interquartile range [IQR]), and categorical variables as frequencies (percentages). Group comparisons were conducted using independent t-tests or Mann-Whitney U tests for continuous variables, according to the normality of the distribution, and chi-square tests for categorical variables.

To examine the association between TyG-BMI and EVA, multivariable logistic regression models were fitted with sequential adjustments for potential confounders, including chronological age, sex, smoking status, drinking status, diabetes status, SBP, LDL-C, and HDL-C. Participants were categorized into TyG-BMI tertiles (T1–T3) to evaluate dose-response relationships. The results are presented as odds ratios (ORs) with 95% confidence intervals (CIs).

Spearman’s rank correlation analysis was performed to assess monotonic associations between TyG-BMI and continuous outcomes, including baPWV, predicted vascular age, age gap, and predicted 10-year CVD risk. Subsequently, multivariable linear regression models were constructed. For models with baPWV as the dependent variable, covariates included chronological age, sex, smoking status, drinking status, diabetes status, SBP, LDL-C, and HDL-C. In contrast, models examining predicted vascular age, age gap, and predicted 10-year CVD risk were not further adjusted for additional covariates, as these FRS-derived metrics already incorporate age, sex, smoking, SBP, and lipid profile through the FRS algorithm itself.

Restricted cubic spline (RCS) analyses were employed to explore potential nonlinear relationships between TyG-BMI and key outcomes. For EVA and baPWV, models were adjusted for chronological age, sex, smoking status, drinking status, diabetes status, SBP, LDL-C, and HDL-C. In contrast, RCS analyses for these FRS-derived metrics (predicted vascular age, age gap, and predicted 10-year CVD risk) were conducted without further adjustment, as the FRS algorithm inherently incorporates the relevant covariates. Receiver operating characteristic (ROC) curves were constructed to evaluate the discriminative performance of TyG‑BMI, TyG index, and BMI for identifying EVA, with performance quantified by the area under the curve (AUC). The DeLong test was performed to compare the AUC of TyG-BMI against those of the TyG index alone and BMI alone, based on predictions from the adjusted models described above.

## Results

### Baseline characteristics EVA versus non-EVA participants

This cross-sectional study included 1,272 participants (127 with EVA and 1,145 without EVA), aged 30 ~ 59 years, for comprehensive analysis (Table 1). Comparative analysis revealed that the EVA group exhibited significantly higher levels of weight, BMI, TC, TG, LDL-C, FPG, HbA1c, SBP, baPWV, the TyG index, and TyG-BMI. This group also contained a higher proportion of males, smokers, drinkers, and individuals with diabetes (all *P* < 0.05). In contrast, chronological age, height, HDL-C and Hcy showed no significant between-group differences. Notably, stratification by TyG-BMI tertiles revealed a stepwise increase in EVA prevalence, rising from 16.54% in the lowest tertile (T1) to 66.14% in the highest tertile (T3).

**Table 1:** Baseline characteristics of the EVA and non-EVA groups

Characteristics	Overall (*n* = 1272)	EVA(*n* = 127)	non-EVA(*n* = 1145)	*P* value
Male, n (%)	1002 (78.77)	121 (95.28)	881(76.94)	< 0.001
Chronological age (years, mean ± SD)	44.03 ± 8.54	44.56 ± 8.53	43.97 ± 8.55	0.465
Smoker, n (%)	246 (19.34)	53 (41.73)	193 (16.86)	< 0.001
Drinker, n (%)	552 (43.40)	77 (60.63)	475(41.48)	< 0.001
Height (cm, mean ± SD)	173.00 ± 8.36	173.91 ± 7.62	172.90 ± 8.43	0.201
Weight (kg, mean ± SD)	76.41 ± 13.36	83.29 ± 12.91	75.65 ± 13.20	< 0.001
BMI (kg/m^2^, mean ± SD)	25.42 ± 3.53	27.55 ± 4.09	25.19 ± 3.38	< 0.001
TC (mmol/L, mean ± SD)	5.21 ± 0.96	5.53 ± 1.59	5.18 ± 0.86	< 0.001
TG (mmol/L, median, [IQR])	1.43 (0.99 ~ 2.07)	2.05 (1.51 ~ 3.07)	1.35 (0.98 ~ 1.98)	< 0.001
LDL-C (mmol/L, mean ± SD)	3.36 ± 0.65	3.66 ± 0.87	3.33 ± 0.62	< 0.001
HDL-C (mmol/L, mean ± SD)	1.30 ± 0.26	1.29 ± 0.24	1.31 ± 0.27	0.432
FPG (mmol/L, mean ± SD)	5.74 ± 1.74	7.57 ± 3.77	5.54 ± 1.18	< 0.001
HbA1c (%, mean ± SD)	5.84 ± 0.89	6.81 ± 1.94	5.74 ± 0.59	< 0.001
Hcy (µmol/L, median, [IQR])	11.81 (9.84 ~ 15.30)	11.95 (10.68 ~ 13.52)	11.76 (9.75 ~ 15.49)	0.490
SBP (mmHg, mean ± SD)	124.73 ± 15.76	141.94 ± 21.09	122.82 ± 13.80	< 0.001
Diabetic, n (%)	138(10.85)	48 (37.80)	90 (7.86)	< 0.001
BaPWV (cm·s^− 1^, mean ± SD)	1300.48 ± 205.11	1667.96 ± 216.82	1259.72 ± 157.82	< 0.001
TyG index (mean ± SD)	8.77 ± 0.61	9.37 ± 0.76	8.71 ± 0.55	< 0.001
TyG-BMI (mean ± SD)	223.87 ± 40.14	259.23 ± 49.80	219.94 ± 36.92	< 0.001
TyG-BMI (tertiles)				
T1 (< 201.34)	424 (33.33)	21 (16.54)	403 (35.20)	< 0.001
T2 (201.34 ~ 236.95)	424 (33.33)	22 (17.32)	402 (35.11)	
T3 (> 236.95)	424 (33.33)	84 (66.14)	340 (29.70)	

### FRS components and FRS-derived indices in EVA versus non-EVA groups

**Fig. 2:**
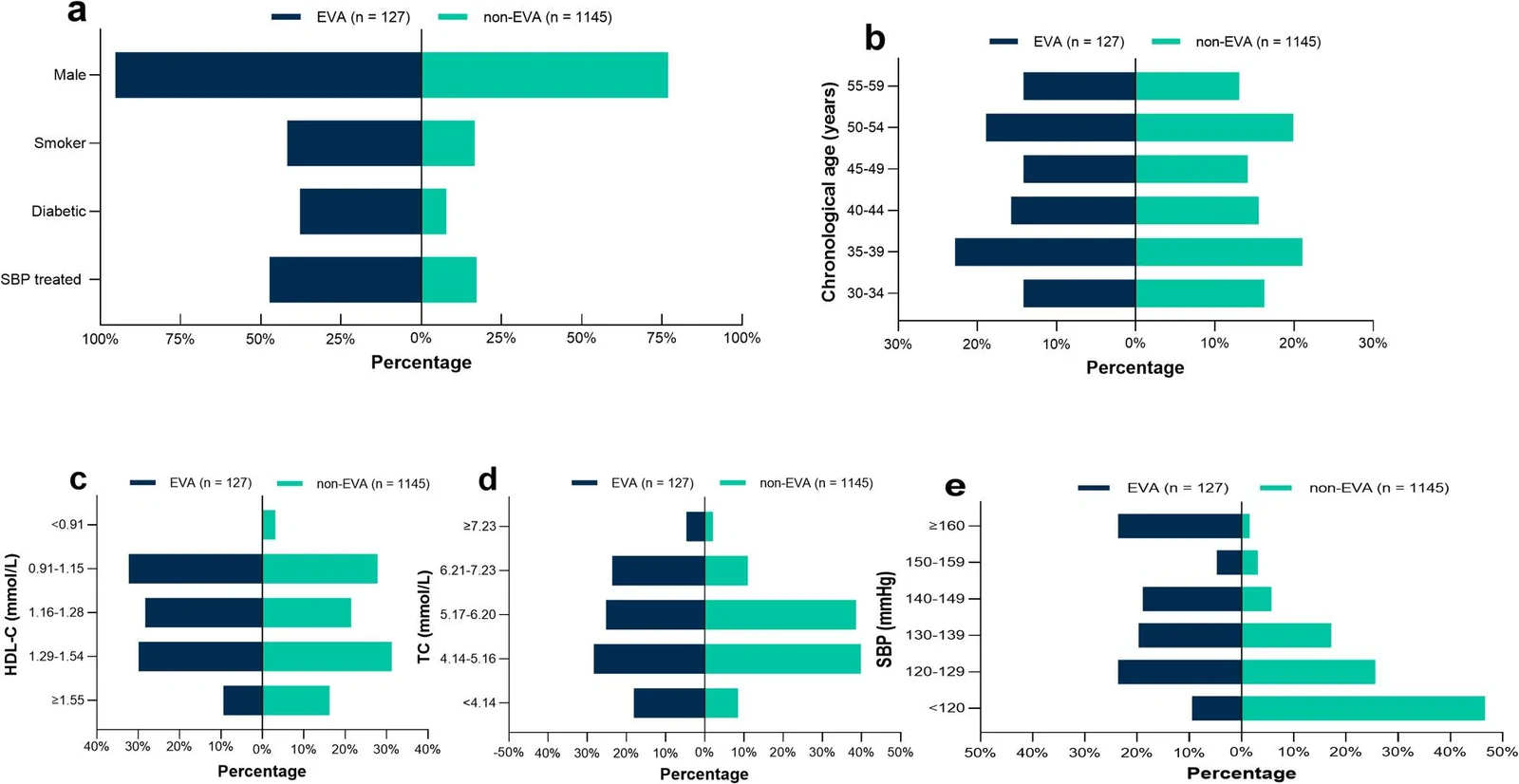
The distribution of components of the FRS between the EVA and non-EVA groups. **a** Sex, smoking status, diabetes status, and SBP treatment. **b** Chronological age. **c** HDL-C levels. **d** TC levels. **e** SBP levels

Table 2; Fig. 2 show the differences in FRS components between the two groups. Chronological age was similar between the two groups after stratification (*P* = 0.986). By contrast, statistically significant differences were detected for all other stratified FRS components: male sex, HDL-C, TC, SBP, antihypertensive treatment history, smoking status and diabetes status (all *P* < 0.05). Based on the FRS, we calculated predicted vascular age, age gap, and predicted 10-year CVD risk. The EVA group manifested significantly higher values across all these parameters (all *P* < 0.001, Table 2).

**Table 2:** Comparison of FRS components and FRS-derived indices between EVA and non-EVA groups

Characteristics	Overall (*n* = 1272)	EVA (*n* = 127)	non-EVA (*n* = 1145)	*P* value
Components of the FRS				
Male, n (%)	1002 (78.77)	121 (95.28)	881(76.94)	< 0.001
Chronological age (years)				
30–34	204 (16.04)	18 (14.17)	186 (16.24)	0.986
35–39	270 (21.23)	29 (22.83)	241 (21.05)	
40–44	198 (15.57)	20 (15.75)	178 (15.55)	
45–49	180 (14.15)	18 (14.17)	162 (14.15)	
50–54	252 (19.81)	24 (18.90)	228 (19.91)	
55–59	168 (13.21)	18 (14.17)	150 (13.10)	
HDL-C (mmol/L)				
≥ 1.55	198 (15.57)	12 (9.45)	186 (16.24)	0.031
1.29–1.54	396 (31.13)	38 (29.92)	358 (31.27)	
1.16–1.28	282 (22.17)	36 (28.35)	246 (21.48)	
0.91–1.15	360 (28.30)	41 (32.28)	319 (27.86)	
< 0.91	36 (2.83)	0 (0)	36 (3.14)	
TC (mmol/L)				
< 4.14	120 (9.43)	23 (18.11)	97 (8.47)	< 0.001
4.14–5.16	492 (38.68)	36 (28.35)	456 (39.83)	
5.17–6.20	474 (37.26)	32 (25.20)	442 (38.60)	
6.21–7.23	156 (12.26)	30 (23.62)	126 (11.00)	
≥ 7.23	30 (2.36)	6 (4.72)	24 (2.10)	
SBP treated history, n (%)	258 (20.28)	60 (47.24)	198 (17.29)	< 0.001
SBP (mmHg)				
< 120	546 (42.92)	12 (9.45)	534 (46.64)	< 0.001
120–129	324 (25.47)	30 (23.62)	294 (25.68)	
130–139	222 (17.45)	25 (19.69)	197 (17.21)	
140–149	90 (7.08)	24 (18.90)	66 (5.76)	
150–159	42 (3.30)	6 (4.72)	36 (3.14)	
≥ 160	48 (3.77)	30 (23.62)	18(1.57)	
Smoker, n (%)	246 (19.34)	53 (41.73)	193 (16.86)	< 0.001
Diabetic, n (%)	138(10.85)	48 (37.80)	90 (7.86)	< 0.001
FRS-derived indices				
Predicted vascular age (years, median, [IQR])	45 (36 ~ 59)	60 (45 ~ 76)	42 (36 ~ 55)	< 0.001
Age gap (years, median, [IQR])	2 (-4 ~ 12.75)	16 (4 ~ 23)	1 (-4 ~ 10)	< 0.001
Predicted 10-year CVD risk (%, median, [IQR])	4.70 (2.33 ~ 10.75)	13.2 (5.6 ~ 21.6)	3.9 (2.3 ~ 9.4)	< 0.001

### Logistic regression analysis for the association between TyG-BMI and EVA

**Fig. 3:**
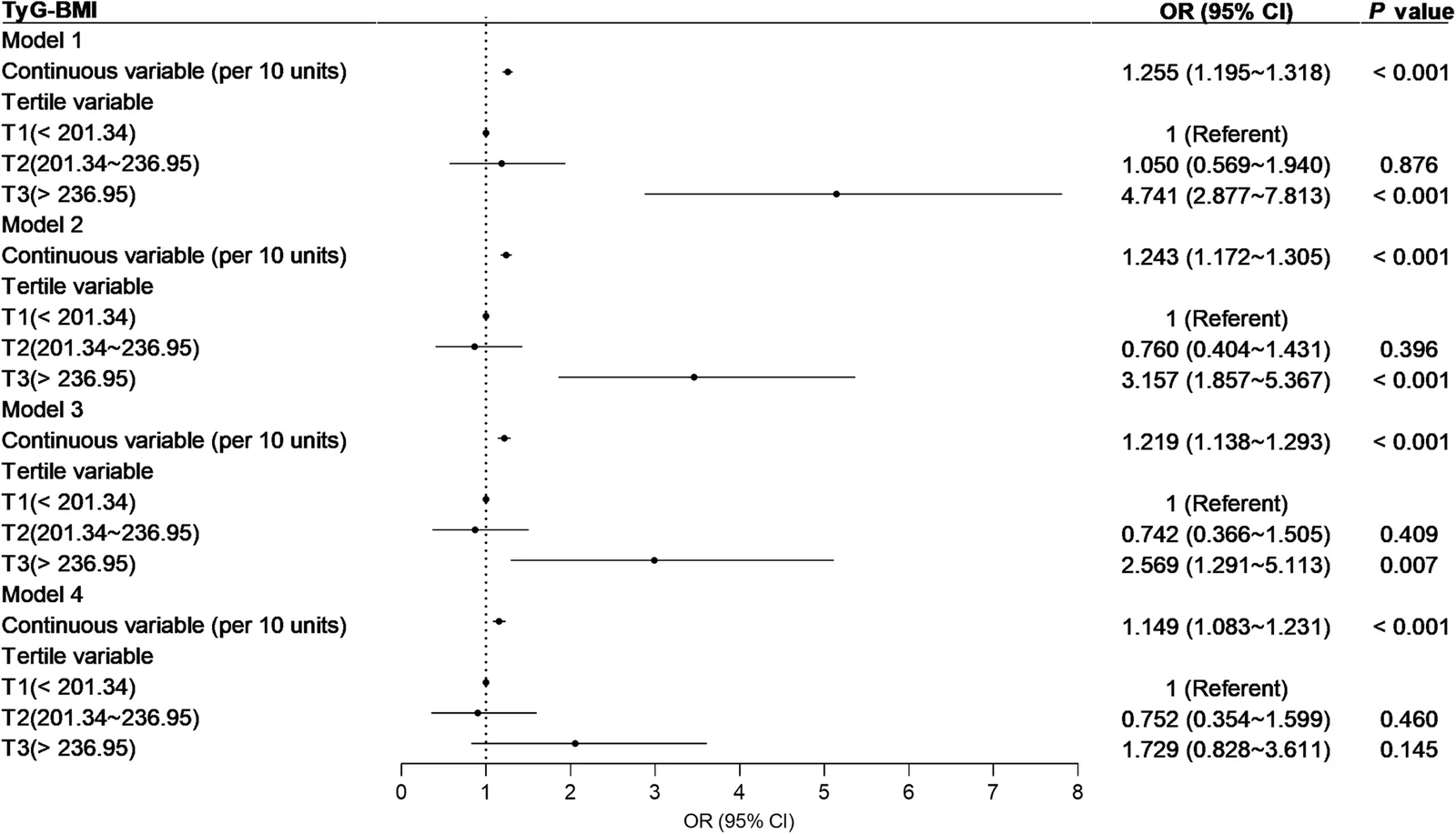
Logistic regression analysis for the relationship between TyG-BMI and EVA. Model 1: Unadjusted. Model 2: Adjusted for chronological age and sex. Model 3: Adjusted for covariates in Model 2 plus smoking status, drinking status, diabetes status, LDL-C, and HDL-C. Model 4: Adjusted for covariates in Model 3 plus SBP

Logistic regression analysis was performed to evaluate the association between TyG-BMI and EVA (Fig. 3). Covariates were sequentially adjusted across four models. Model 3, which included chronological age, sex, smoking status, drinking status, diabetes status, LDL-C, and HDL-C, served as the primary multivariable model. Model 4 was further adjusted for SBP. When TyG-BMI was analyzed as a continuous variable, each 10-unit increment in TyG-BMI was significantly associated with an increased risk of EVA across all models: OR 1.255 (95% CI: 1.195 ~ 1.318) in Model 1, 1.243 (1.172 ~ 1.305) in Model 2, 1.219 (1.138 ~ 1.293) in Model 3, and 1.149 (1.083 ~ 1.231) in Model 4. When TyG-BMI was categorized into tertiles, T2 did not differ significantly from T1 in any model. Compared with T1, T3 showed a significantly elevated risk of EVA in Models 1–3, but this association was attenuated and became non-significant in Model 4.

### Spearman correlation analyses between TyG-BMI and baPWV as well as FRS-derived indices

As demonstrated in Table 3, TyG-BMI exhibited significant positive correlations with baPWV (rcorrelation coefficient [r] = 0.245), predicted vascular age (*r* = 0.328), age gap (*r* = 0.528), and predicted 10-year CVD risk (*r* = 0.356), all *P* < 0.001.

**Table 3:** Spearman correlation analyses between TyG-BMI and baPWV as well as FRS-derived indices

TyG-BMI	BaPWV	Predicted vascular age	Age gap	Predicted 10-year CVD risk
*r*	0.245	0.328	0.528	0.356
*P* value	< 0.001	< 0.001	< 0.001	< 0.001

### Linear regression analyses of the associations of TyG-BMI with baPWV and FRS-derived indices

**Fig. 4:**
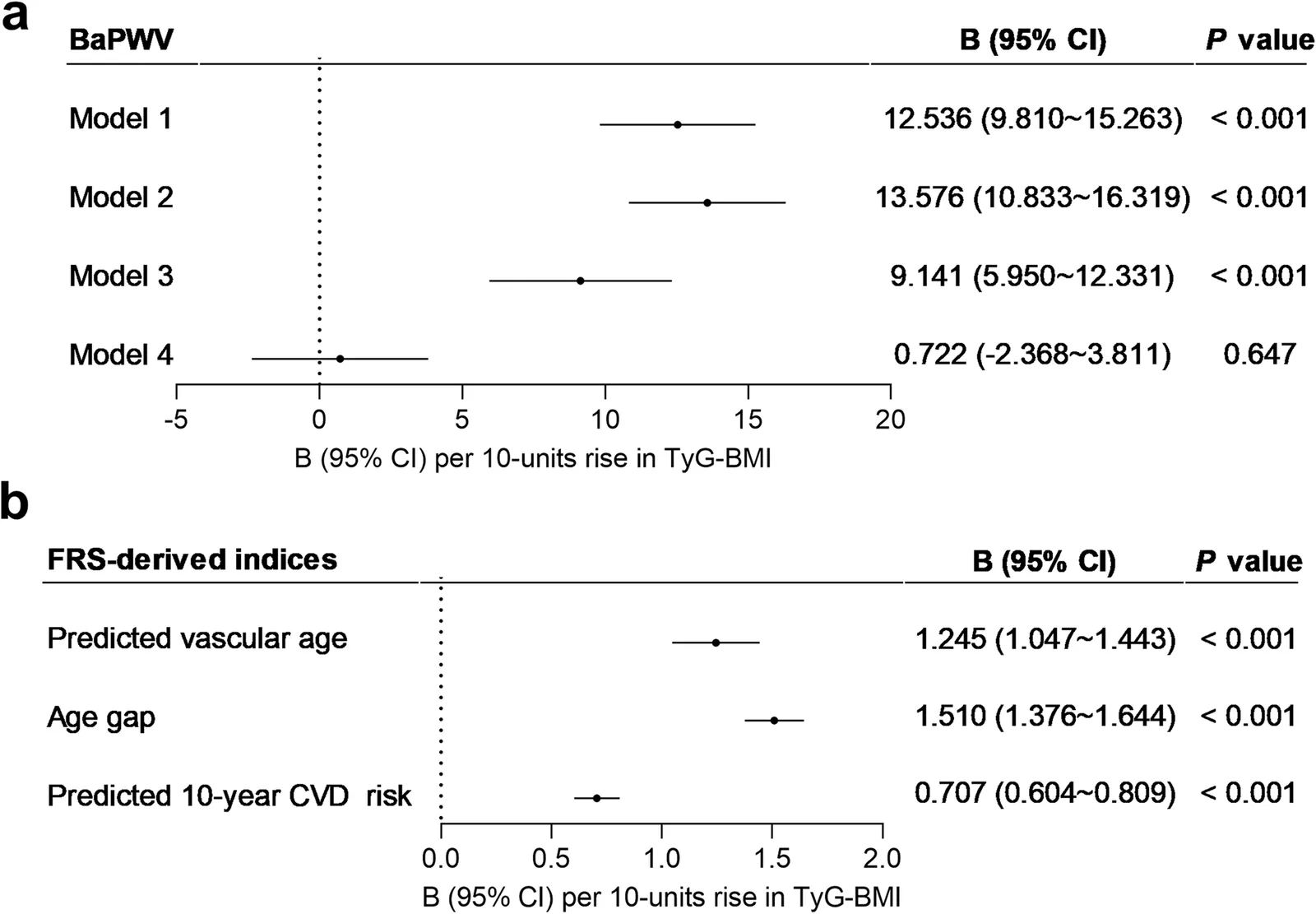
Linear regression analyses of the associations of TyG-BMI with baPWV and FRS-derived indices. **a**​ Association with baPWV. **b**​ Association with FRS-derived indices (predicted vascular age, age gap, and predicted 10-year CVD risk). Model 1: Unadjusted. Model 2: Adjusted for chronological age and sex. Model 3: Adjusted for covariates in Model 2 plus smoking status, drinking status, diabetes status, LDL-C, and HDL-C. Model 4: Adjusted for covariates in Model 3 plus SBP

Linear regression analysis was conducted to explore the association between the TyG-BMI and baPWV. As illustrated in Fig. 4a, TyG-BMI exhibited a significant positive correlation with baPWV in both the unadjusted crude model and partially adjusted models. In the unadjusted Model 1, every 10-unit increment in TyG-BMI was associated with a significant elevation of 12.536 cm/s in baPWV (95% CI: 9.810 ~ 15.263, *P* < 0.001). This positive correlation remained stable in Model 2 adjusted for conventional confounding factors (chronological age and sex), with a regression coefficient (B) of 13.576 (95% CI: 10.833 ~ 16.319, *P* < 0.001). After further adjustment for multiple clinical covariates in Model 3, the significant positive association between TyG-BMI and baPWV persisted (B = 9.141, 95% CI: 5.950 ~ 12.331, *P* < 0.001). Notably, in the fully adjusted Model 4 with additional adjustment for SBP, this association was no longer statistically significant, with a near-zero B of 0.722 (95% CI: −2.368 ~ 3.811, *P* = 0.647).

In Fig. 4b, for the FRS-derived indicators (namely, predicted vascular age, age gap, and predicted 10-year CVD risk), TyG-BMI showed significant positive associations with all three outcomes in unadjusted models (all *P* < 0.001). Considering the inherent collinearity between TyG-BMI and conventional risk factors incorporated into the FRS algorithm, these analyses were retained in an unadjusted state to avoid excessive statistical correction and ensure the reliability of the results.

### RCS analyses of the associations of TyG-BMI with EVA, baPWV and FRS-derived indices

**Fig. 5:**
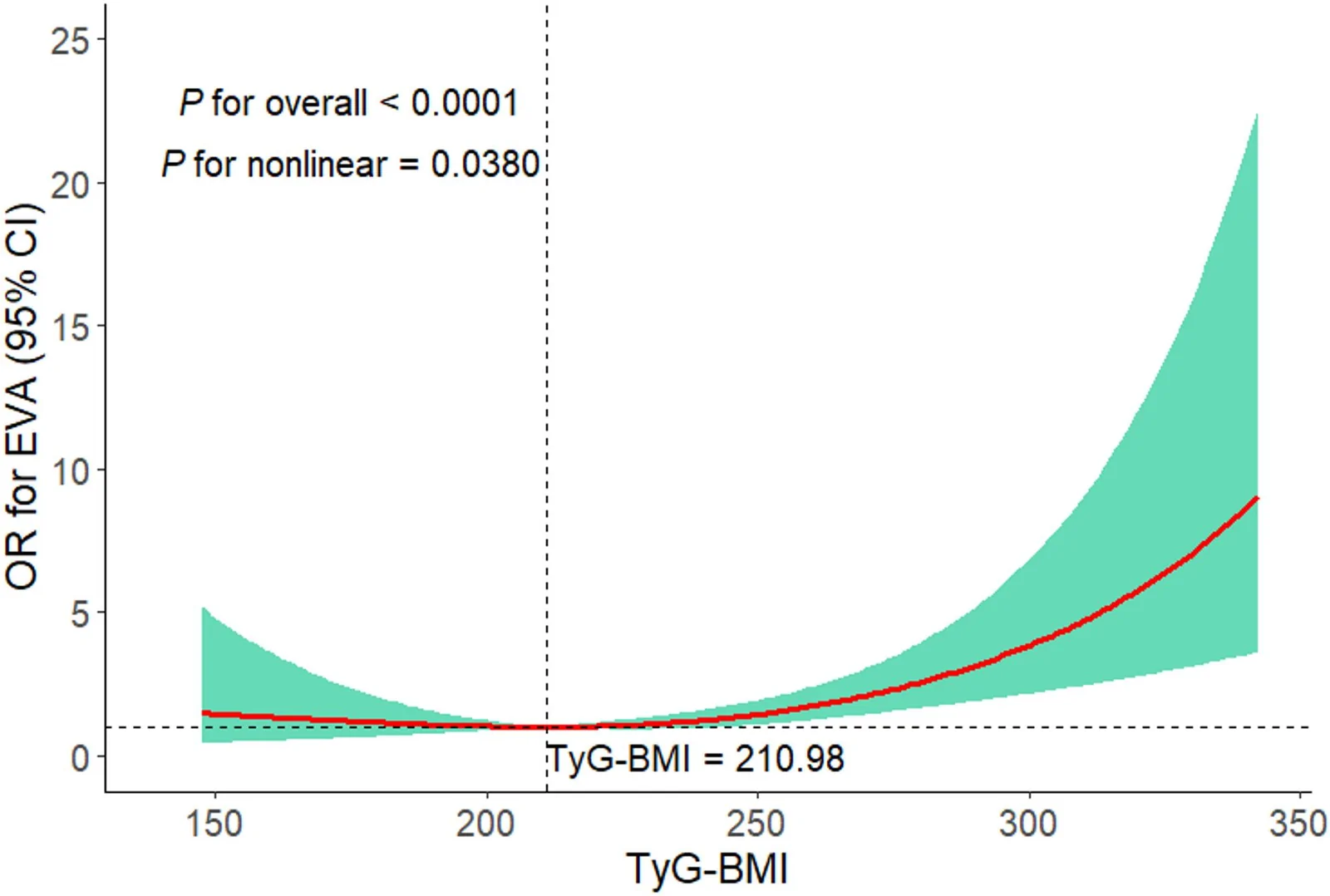
RCS analysis of the association between TyG-BMI and EVA. The model was adjusted for chronological age, sex, smoking status, drinking status, diabetes status, SBP, LDL-C, and HDL-C (Model 4)

RCS analysis was performed to explore the potential non-linear association between TyG-BMI and EVA. As shown in Fig. 5, the relationship between TyG-BMI and EVA risk exhibited a statistically significant non-linear pattern (*P* for non-linearity = 0.0380), with an overall significant association (*P* for overall < 0.0001). Below the turning point (TyG-BMI = 210.98), the curve demonstrated a numerical downward trend; however, the 95% confidence interval crossed the null value of 1, indicating that this decline was not statistically significant. Conversely, beyond this turning point, the OR for EVA increased steeply, suggesting a sharp elevation in risk with higher TyG-BMI levels.

**Fig. 6:**
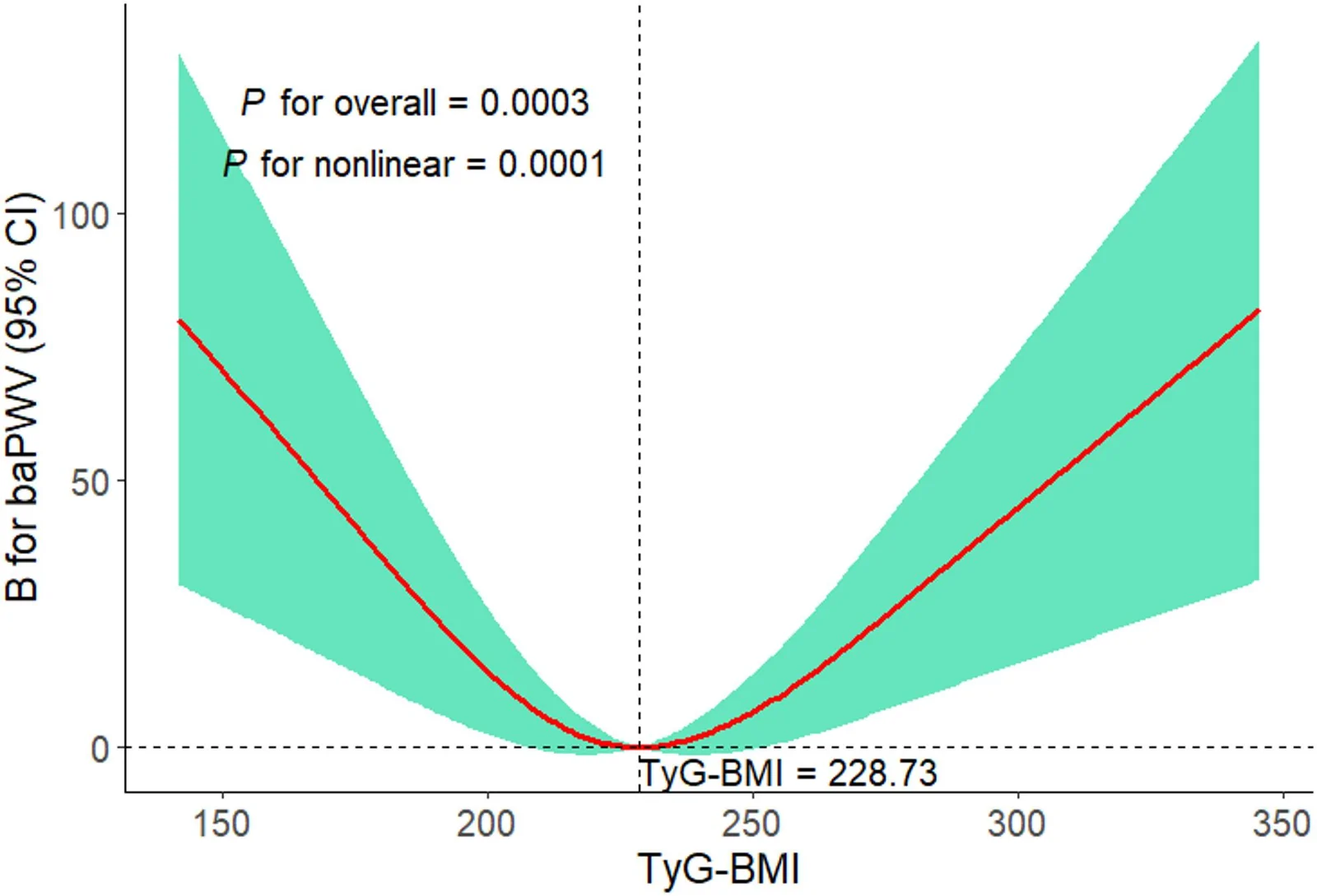
RCS analysis of the association between TyG-BMI and baPWV. The model was adjusted for chronological age, sex, smoking status, drinking status, diabetes status, SBP, LDL-C, and HDL-C (Model 4)

RCS analysis revealed a significant U-shaped association between the TyG-BMI index and baPWV (*P* for overall = 0.0003, *P* for nonlinear = 0.0001), with TyG-BMI = 228.73 serving as the reference point (Fig. 6). The B decreased progressively as TyG-BMI approached the turning point, followed by a steady increase thereafter.

**Fig. 7:**
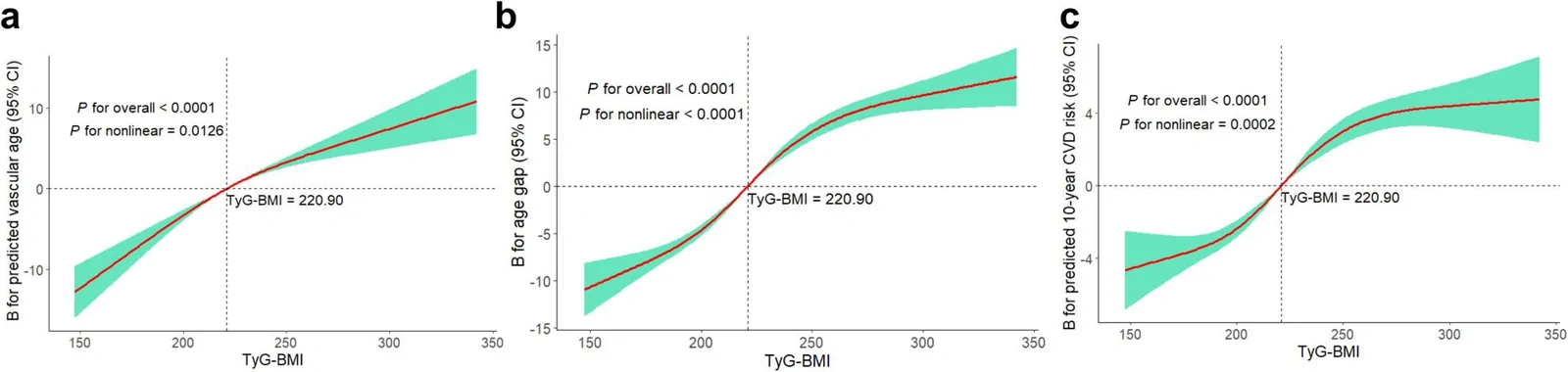
RCS analysis of the association between TyG-BMI and FRS-derived indices. **a**-**c**: Model 1(unadjusted)

In Fig. 7, unadjusted RCS analyses demonstrated significant non-linear relationships between TyG-BMI and the three FRS-derived indicators (predicted vascular age, age gap, and predicted 10-year CVD risk). The overall and non-linearity test results were as follows: *P*_overall_ < 0.0001 for all indicators; *P*_nonlinear_ = 0.0126 for predicted vascular age, *P*_nonlinear_ < 0.0001for age gap, and *P*_nonlinear_ = 0.0002 for predicted 10-year CVD risk. A unified turning point was detected at TyG-BMI = 220.90. Below this threshold, TyG-BMI was negatively correlated with all three indices. When TyG-BMI exceeded 220.90, the association turned positive. For predicted vascular age, the rising trend gradually slowed down at higher TyG-BMI levels. By contrast, age gap and predicted 10-year CVD risk presented an obvious plateau effect after the turning point. These findings merely illustrate general trends and should be interpreted with caution due to potential mathematical coupling.

### ROC curve of TyG-BMI for predicting EVA

**Fig. 8:**
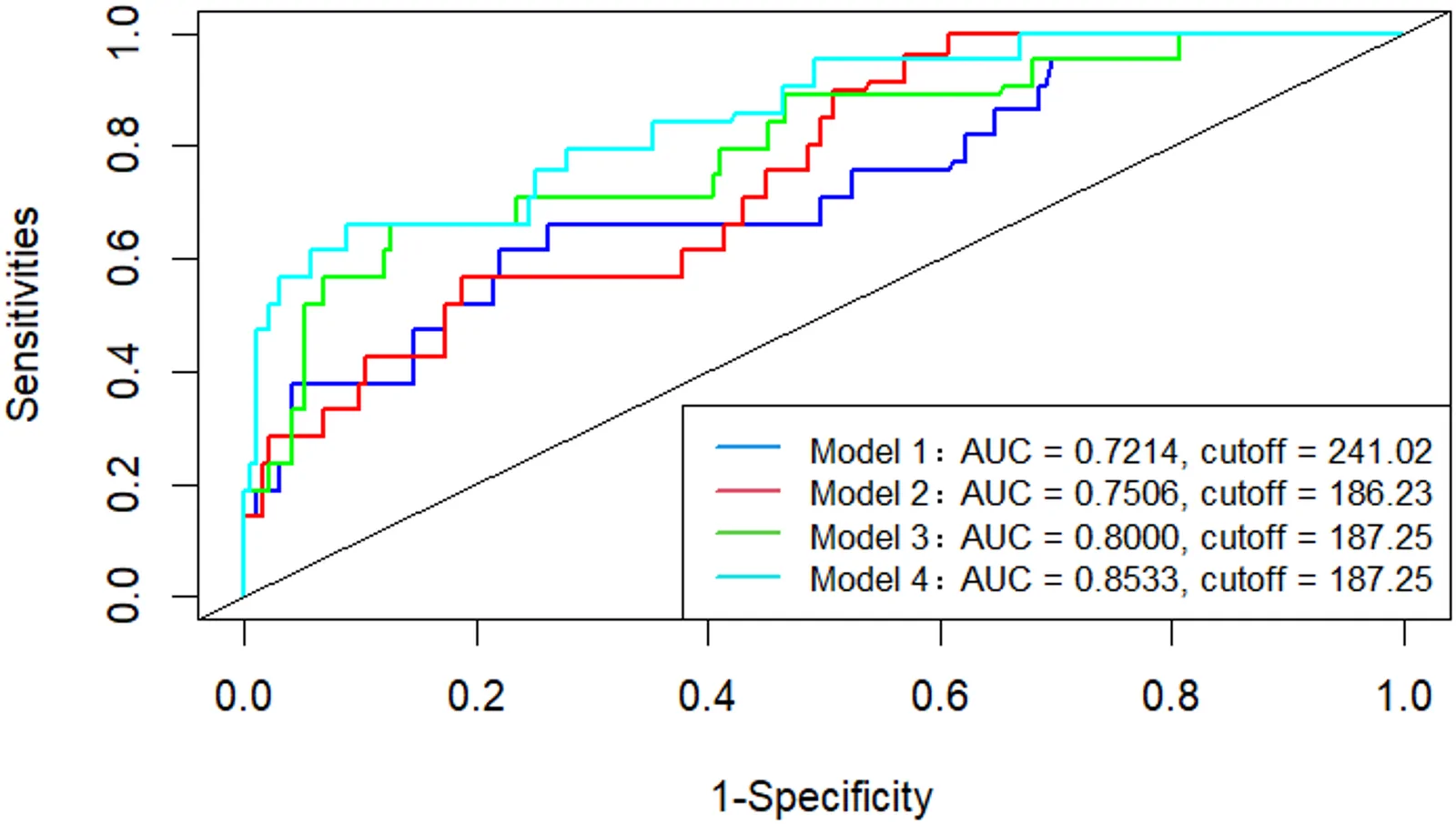
ROC curves of TyG-BMI for predicting EVA. Model 1: Unadjusted. Model 2: Adjusted for chronological age and sex. Model 3: Adjusted for covariates in Model 2 plus smoking status, drinking status, diabetes status, LDL-C, and HDL-C. Model 4: Adjusted for covariates in Model 3 plus SBP

Hierarchical ROC curve analyses were performed to evaluate the discriminatory capacity of TyG-BMI for EVA (Fig. 8), with explicit differentiation between unadjusted single-marker performance (Model 1, raw TyG-BMI without any covariate correction) and sequentially multivariable-adjusted model performance (Models 2–4). All optimal classification cutoffs were determined via the maximum Youden index, with complete diagnostic metrics presented as follows: Model 1 (unadjusted): AUC = 0.7214 (95% CI: 0.6720–0.7708, *P* < 0.001), cutoff = 241.02; Model 2 (adjusted for age and sex): AUC = 0.7506 (95% CI: 0.7102–0.7910, *P* < 0.001), cutoff = 186.23; Model 3 (Model 2 covariates plus smoking, drinking, diabetes, LDL-C and HDL-C): AUC = 0.8000 (95% CI: 0.7563–0.8437, *P* < 0.001), cutoff = 187.25; Model 4 (fully adjusted with additional SBP): AUC = 0.8533 (95% CI: 0.8173–0.8892, *P* < 0.001), cutoff = 187.25, with a corresponding sensitivity of 66.14% and specificity of 91.09%. Stepwise adjustment for confounders steadily elevated the AUC value, indicating improved predictive performance. Notably, further adjusting for SBP raised the overall discriminatory power but did not change the cutoff shared by Model 3 and Model 4.

**Fig. 9:**
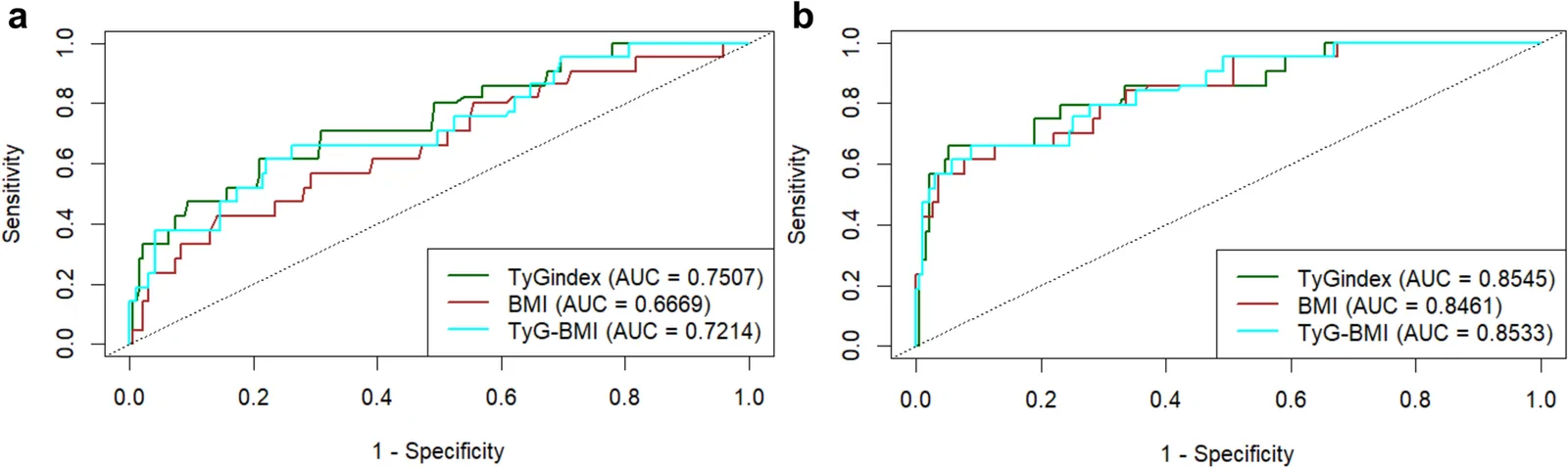
ROC curves of TyGindex, BMI, and TyG-BMI for predicting EVA. **a** Model 1(unadjusted); **b**: Model 4 (adjusted for chronological age, sex, smoking status, drinking status, SBP, diabetes status, LDL-C, and HDL-C)

ROC curve analysis (Fig. 9) evaluated the predictive performance of TyG index, BMI, and TyG-BMI for EVA. In the unadjusted model, the AUC values were 0.7507, 0.6669, and 0.7214 for TyG index, BMI, and TyG-BMI, respectively. TyG index and TyG-BMI showed comparable predictive performance (*P* = 0.1624), while TyG-BMI significantly outperformed BMI (*P* < 0.001). In the fully adjusted model (Model 4), the corresponding AUCs were 0.8545, 0.846, and 0.8533. The comparable performance between TyG index and TyG-BMI persisted (*P* = 0.7718), and TyG-BMI remained significantly superior to BMI (*P* = 0.0172).

## Discussion

In this cross-sectional study of young and middle-aged Chinese adults, we observed a significant positive association between TyG-BMI and the risk of EVA, robust after full adjustment for confounders. Importantly, the relationship exhibited a distinct non-linear pattern. While the risk showed a slight non-significant decreasing trend within the lower TyG-BMI range (below 210.98), it increased sharply once values exceeded this turning point. These findings suggest that TyG-BMI may serve as a practical indicator for assessing EVA risk, particularly in individuals with elevated metabolic burden.

Vascular aging represents a complex biological process marked by progressive structural and functional deterioration, typically presenting as subclinical vascular damage before overt clinical events occur [20]. This process begins during fetal development and persists throughout the lifespan, with its progression potentially accelerated by multiple pro-aging factors, particularly cardiovascular risk factors, thereby increasing susceptibility to EVA [21]. To objectively quantify EVA, we utilized baPWV, a well-validated measure of arterial stiffness that captures these subclinical changes. EVA was defined as a baPWV value exceeding the 90th percentile within the corresponding 10-year age group [9, 10].

The TyG index is a well-established surrogate marker of IR. Accumulating evidence links elevated TyG levels to adverse vascular aging outcomes [22–24]. For instance, a large Chinese retrospective cohort reported that higher TyG levels predicted an increased risk of arterial stiffness after rigorous multivariable adjustment [24]. Similarly, BMI as a conventional measure of adiposity is also implicated in vascular senescence [25–27]. Longitudinal data from the German KiGGS cohort (KiGGS; German Health Interview and Examination Survey for Children and Adolescents) demonstrated that childhood obesity substantially contributed to subclinical atherosclerosis and arterial stiffness in early adulthood [26]. However, both TyG and BMI have inherent limitations when used as independent predictors. TyG primarily reflects glycometabolic dysfunction but does not capture adiposity-related risk, whereas BMI fails to account for underlying metabolic abnormalities such as IR. The composite biomarker TyG-BMI, calculated as the product of the TyG index and BMI, simultaneously incorporates glycometabolic and adiposity-related information.

TyG-BMI has emerged as a promising indicator of IR [12]. Given the well-established associations of both TyG and BMI with vascular aging and the central role of IR in vascular pathophysiology, it is biologically plausible that TyG-BMI is positively associated with EVA risk. Although direct evidence linking TyG-BMI to EVA remains limited, accumulating studies have demonstrated robust associations between TyG-BMI and cardiovascular diseases, which represent advanced manifestations of vascular aging [17, 28–30]. A recent meta-analysis including more than 870,000 participants reported significantly higher risks of cardiovascular disease, coronary artery disease, and stroke among individuals with elevated TyG-BMI levels [28]. Similar associations have been observed in population-based surveys and clinical cohorts, further supporting the potential relevance of TyG-BMI in cardiovascular risk stratification [17, 29, 30]. Therefore, elucidating the specific relationship between TyG-BMI and EVA constitutes the primary aim of this study.

The present study found a consistent positive association between TyG-BMI and EVA risk in adults aged 30 ~ 59. This association remained statistically significant across all adjustment models, suggesting that TyG-BMI may serve as an independent predictor of EVA beyond conventional cardiovascular risk factors.

The biological mechanisms underlying the association between TyG-BMI and EVA are likely multifactorial and remain incompletely elucidated. As a composite marker of IR, TyG-BMI captures both adiposity and glycometabolic dysfunction. IR, a core feature of metabolic syndrome, has been implicated in dyslipidemia, endothelial dysfunction, vascular inflammation, and atherosclerotic progression, all of which contribute to vascular aging [31]. IR promotes hypertriglyceridemia, reduces high-density lipoprotein cholesterol levels, and increases the production of small dense low-density lipoprotein particles, thereby exacerbating endothelial injury [32]. In addition, IR impairs endothelial function through increased oxidative stress, activation of proinflammatory signaling pathways, reduced nitric oxide bioavailability, and disruption of cyclic guanosine monophosphate signaling in vascular smooth muscle cells. These alterations collectively lead to reduced arterial compliance, increased arterial stiffness, and accelerated vascular aging [33–35].

Furthermore, our investigation analyzed the TyG-BMI as categorical variables in relation to EVA risk, thus reducing information loss and quantifying their relationship. In this cross-sectional study of adults aged 30 ~ 59 years, TyG-BMI was initially categorized into tertiles (< 201.34, 201.34 ~ 236.95, > 236.95). While the highest tertile (T3) showed an elevated risk of EVA in crude models, this association attenuated to non-significance after adjusting for SBP in Model 4. Rather than negating the role of TyG-BMI, this attenuation highlights the limitation of arbitrary categorization in capturing complex dose-response relationships. To address this, RCS regression was employed. The RCS analysis revealed a significant non-linear association (*P* for non-linear = 0.0380), identifying a distinct inflection point at TyG-BMI 210.98. The risk of EVA showed a slight non-significant decreasing trend below this turning point but increased sharply thereafter, confirming that vascular hazard is concentrated exclusively above this cut-off. Mechanistically, this reflects the transition from compensated metabolic stress to overt vascular injury driven by insulin resistance and adiposity. Crucially, this continuous modeling clarifies the categorical findings. The middle tertile (T2) straddles the 210.98 turning point, mixing individuals on the plateau with those on the ascending limb, thereby diluting any detectable contrast against T1. For T3, although the RCS demonstrates a steep risk gradient beyond 210.98, the adjustment for SBP—a dominant hemodynamic correlate—absorbed the shared variance, rendering the specific contrast between T3 and T1 statistically unstable. This does not imply that high TyG-BMI is harmless. Instead, SBP may act as a final common pathway through which metabolic excess exerts its damage. In summary, RCS analysis identified an inflection point at 210.98, beyond which the association between TyG-BMI and EVA risk appeared to strengthen. These findings highlight the importance of considering non-linear relationships in future research, rather than relying solely on linear assumptions or broad categorical comparisons.

These findings are in line with previous studies reporting nonlinear relationships between TyG-BMI and cardiovascular outcomes. A previous analysis of the National Health and Nutrition Examination Survey (NHANES) data revealed a nonlinear association between TyG-BMI and CVD. No significant association was detected below the threshold of 260, and each 10-unit rise in TyG-BMI above this cutoff increased CVD odds by 2.4% [36]. Although some investigations have reported linear associations [28]. The discrepancies across studies may be attributable to differences in study design, population characteristics, and outcome definitions. Importantly, our focus on subclinical vascular aging markers in a relatively young population may partly explain the observed threshold effects, as metabolic disturbances may exert more pronounced nonlinear influences during the early stages of vascular damage.

The identification of these nonlinear associations has important clinical implications, suggesting that TyG-BMI may be particularly useful for identifying individuals at high risk of EVA who may benefit most from targeted preventive interventions. Longitudinal studies are warranted to confirm these findings and to further elucidate the temporal and mechanistic relationships between TyG-BMI and EVA.

To extend our findings regarding TyG-BMI and EVA, we evaluated its dose-response relationship with arterial stiffness, a fundamental precursor to structural vascular damage. Linear regression revealed a robust positive association between TyG-BMI and baPWV, which was completely attenuated to non-significance after adjusting for SBP (B = 0.722, *P* = 0.647). This underscores that hemodynamic load acts as the primary mediator linking metabolic dysfunction to arterial stiffness. Complementing this, RCS analysis identified a significant U-shaped association (*P* for non-linear < 0.001), with a turning point at TyG-BMI = 228.73, which indicated that both extremes of metabolic status were significantly associated with increased arterial stiffness. Integrating these findings, the U-shaped dose-response explains the fragility of the linear model: maintaining TyG-BMI within the narrow range surrounding the 228.73 turning point appears most critical for vascular health, as the risk escalates significantly once this threshold is exceeded, irrespective of SBP. This U-shaped relationship can be explained by the dual composition of TyG-BMI. High TyG-BMI represents concurrent IR and abdominal obesity, which jointly trigger inflammation and metabolic disorders and further accelerate arteriosclerosis [37]. By contrast, low TyG-BMI may suggest inadequate nutrition, hypometabolism and low adiposity, a phenotype closely linked to sarcopenia and malnutrition. Notably, a similar non-linear pattern has also been documented for another TyG-derived index. A prior study reported a potential U-shaped association between cumulative exposure to TyG-WHtR and both changes in baPWV and its annual rate of change [38].

Although TyG-BMI exhibited non-linear associations with both baPWV and EVA, their dose-response curves differed due to distinct outcome metrics and definitions. As a continuous variable, baPWV showed a gradual, symmetric U-shaped trend across all TyG-BMI values, with the turning point representing the optimal metabolic state. As a binary outcome defined by the age-stratified 90th percentile of baPWV, EVA targets the upper tail of arterial stiffness distribution. Accordingly, its curve mainly reflects the rapid increase in odds of extreme arterial stiffness when TyG-BMI exceeds the vulnerable range. Before the inflection point, the EVA RCS curve shows a slight non-significant decreasing trend due to sparse data at the 90th percentile, followed by a steep rise thereafter. In contrast, the baPWV curve presents a smooth, continuous trend. BaPWV indicates arterial stiffness severity, while EVA reflects the risk of abnormal arterial stiffness. Collectively, these results confirm that TyG-BMI is associated with both arterial stiffening and EVA phenotypes.

As a secondary outcome, vascular age was estimated using the FRS, a validated composite index that integrates multiple cardiovascular risk factors into a single predictive measure, including sex, age, high-density lipoprotein cholesterol, total cholesterol, systolic blood pressure (treated or untreated), smoking status, and diabetes status [11]. We evaluated the association between TyG-BMI and FRS-derived indicators of long-term cardiovascular risk. Unadjusted restricted cubic spline analyses revealed significant non-linear associations between TyG-BMI and Framingham-based metrics (predicted vascular age, age gap, and predicted 10-year CVD risk). A unified turning point at TyG-BMI = 220.90 was identified across all outcomes, below which TyG-BMI appeared negatively associated and above which it turned positive. However, these findings should be interpreted as descriptive trends. Due to the intrinsic multicollinearity between TyG-BMI and the constituent variables of the FRS, multivariable adjustment was deemed inappropriate as it would lead to over-adjustment bias and spurious null findings. Therefore, although the consistent inflection at 220.90 indicates a potential threshold effect in overall cardiovascular risk, we cannot conclude that TyG-BMI exerts effects independently of conventional risk factors included in the FRS.

In addition, ROC curve analyses demonstrated that TyG-BMI exhibited good discriminatory performance for identifying EVA after adjustment for confounding factors. Previous studies have reported moderate predictive value of TyG-BMI for cardiovascular outcomes in different clinical settings [39–42]. In the present study, the empirical TyG-BMI cutoff for EVA risk classification decreased after adjustment for confounders, suggesting that maintaining TyG-BMI below this threshold may inform targeted EVA risk reduction strategies. Notably, although the adjustment for SBP in Model 4 significantly elevated the AUC compared to Model 3 (0.8533 vs. 0.8000), the optimal cutoff value remained stable at 187.25. This suggests that the added predictive information from SBP enhanced the overall discriminative ability without shifting the clinical decision threshold. The ROC-derived cutoff was 187.25 (Model 4), lower than the mean TyG-BMI among non-EVA participants (219.94 ± 36.92), whereas the RCS inflection point was 210.98. This divergence arises from their distinct analytical objectives: the ROC cutoff is optimized for binary classification of EVA risk, while the RCS inflection point denotes the threshold at which the association between TyG-BMI and EVA risk strengthens significantly, potentially aligning with physiological shifts in vascular aging. Consistent with our previous conclusion, 210.98 should be interpreted as a statistical reference point for identifying elevated EVA risk, while the 187.25 cutoff is strictly cohort-specific and not generalizable beyond this study.

In the fully adjusted model (Model 4), ROC analysis identified TyG-BMI as a favorable marker for EVA risk, with an AUC of 0.8533. Pairwise comparisons of discriminatory performance revealed that TyG-BMI significantly outperformed BMI alone (AUC = 0.8461, *P* = 0.017), but showed no statistically significant advantage over the TyG index (AUC = 0.8545, *P* = 0.772). These patterns may reflect that integrating adiposity and IR captures complementary risk information relative to BMI alone; however, the comparable performance to TyG suggests IR carries the majority of the predictive signal, with BMI contributing limited incremental value in this context. Importantly, TyG-BMI should be interpreted as a pragmatic tool for EVA risk stratification rather than a diagnostic biomarker, and its clinical utility requires validation in prospective cohorts.

From a public health perspective, our findings suggest that TyG-BMI may serve as a low-cost, readily accessible metric for large-scale population-level risk assessment of EVA. Given that TyG-BMI can be calculated from routine clinical laboratory data, it may be well suited for preliminary community and occupational health surveys to identify subgroups at elevated vascular aging risk and conduct initial targeted risk stratification.

This study has several strengths. First, TyG-BMI was examined in both continuous and categorical forms, allowing for comprehensive evaluation of dose–response relationships while minimizing information loss. Second, by incorporating nonlinear analytical approaches, this study is, to our knowledge, the first to report a nonlinear association between TyG-BMI and EVA. Third, multiple sensitivity analyses were conducted to assess the stability and robustness of the findings. Finally, the analysis of this relatively young population underscores the value of detecting early vascular abnormalities and conducting targeted health management in this group.

Several limitations should also be acknowledged. First, the cross-sectional study design prevents definitive causal inference and fails to establish the temporal sequence between TyG-BMI and EVA. Second, although core clinical confounders were fully adjusted, data on pack-years of smoking, physical activity, dietary patterns and socioeconomic status were unavailable. Unmeasured adverse factors (e.g., heavy smoking, sedentary behavior, poor diet and lower socioeconomic status) may introduce residual confounding and slightly overestimate the observed association. Third, our cohort consisted of 30 ~ 59-year-old health check-up participants from a single center, with females accounting for only 21.23% of all subjects, limiting extrapolation to other age groups, female-dominated populations, residents from other geographic regions and non-Asian ethnic cohorts. Future multicenter prospective studies collecting complete lifestyle data are needed to verify the correlational relationship between TyG-BMI and EVA.

## Conclusion

This cross-sectional study found a significant and independent association between elevated TyG-BMI levels and EVA risk among young and middle-aged adults. RCS analysis revealed a nonlinear relationship with a turning point at 210.98, beyond which risk increased significantly, while the association was nonsignificant below it. Although TyG-BMI may serve as a readily accessible indicator for risk stratification, the inflection point should be interpreted as a statistical reference rather than a validated clinical cutoff. Further prospective, multi-center studies are warranted to confirm these findings before clinical translation.

## Data Availability

The datasets used and/or analysed during the current study are available from the corresponding author on reasonable request.

## Abbreviations

EVA: Early vascular aging
CVD: Cardiovascular disease
IR: Insulin resistance
TyG-BMI: Triglyceride-glucose body mass index
TyG: Triglyceride-glucose
BMI: Body mass index
baPWV: Brachial-ankle pulse wave velocity
RCS: Restricted cubic spline
FRS: Framingham Risk Score
ROC: Receiver operating characteristic
OR: Odds ratio
CI: Confidence interval
AUC: Area under the curve
DM: Diabetes mellitus
HDL-C: High-density lipoprotein cholesterol
TC: Total cholesterol
SBP: Systolic blood pressure
TG: Triglyceride
LDL-C: Low-density lipoprotein cholesterol
FPG: Fasting plasma glucose
HbA1c: Glycosylated hemoglobin
Hcy: Homocysteine
SD: Standard deviation
IQR: Interquartile range
r: Correlation coefficient
B: Regression coefficient
KiGGS: German Health Interview and Examination Survey for Children and Adolescents
NHANES: National Health and Nutrition Examination Survey
